# Field Deployment of a Mobile Biosafety Laboratory Reveals the Co-Circulation of Dengue Viruses Serotype 1 and Serotype 2 in Louga City, Senegal, 2017

**DOI:** 10.1155/2021/8817987

**Published:** 2021-03-31

**Authors:** Idrissa Dieng, Maryam Diarra, Moussa Moïse Diagne, Martin Faye, Marie Henriette Dior Ndione, Yamar Ba, Mamadou Diop, El Hadji Ndiaye, Paolo Marinho de Andrade Zanotto, Boly Diop, Mamadou Ndiaye, Abdoulaye Bousso, Ndongo Dia, Mawlouth Diallo, Aliou Barry, Gamou Fall, Cheikh Loucoubar, Amadou Alpha Sall, Ousmane Faye, Oumar Faye

**Affiliations:** ^1^Arboviruses and Haemorrhagic Fever Viruses Unit, Virology Department, Institut Pasteur de Dakar, Dakar 220, Senegal; ^2^Epidemiology, Clinical Research and Data Science Unit, Institut Pasteur de Dakar, Dakar 220, Senegal; ^3^Medical Entomology Unit, Institut Pasteur de Dakar, Dakar, 36 Avenue Pasteur, Senegal; ^4^Laboratory of Molecular Evolution and Bioinformatics, Department of Microbiology, Biomedical Sciences Institute, University of SP, SP, Brazil; ^5^Prevention Department, Ministry of Health, Dakar, Senegal

## Abstract

Dengue virus (DENV) is the most prevalent arboviral threat worldwide. This virus belonging to genus *Flavivirus*, *Flaviviridae* family, is responsible for a wide spectrum of clinical manifestations, ranging from asymptomatic or mild febrile illness (dengue fever) to life-threatening infections (severe dengue). Many sporadic cases and outbreaks have occurred in Senegal since 1970. Nevertheless, this article describes a field investigation of suspected dengue cases, between 05 September 2017 and 17 December 2017 made possible by the deployment of a Mobile Biosafety Laboratory (MBS-Lab). Overall, 960 human sera were collected and tested in the field for the presence of viral RNA by real-time RT-PCR. Serotyping, sequencing of complete *E* gene, and phylogenetic analysis were also performed. Out of 960 suspected cases, 131 were confirmed dengue cases. The majority of confirmed cases were from Louga community. Serotyping revealed two serotypes, Dengue 1 (100/104; 96, 15%) and Dengue 2 (04/104; 3, 84%). Phylogenetic analysis of the sequences obtained indicated that the Dengue 1 strain was closely related to strains isolated, respectively, in Singapore (Asia) in 2013 (KX380803.1) outbreak and it cocirculated with a Dengue 2 strain closely related to strains from a Burkina Faso dengue outbreak in 2016 (KY62776.1). Our results showed the co-circulation of two dengue virus serotypes during a single outbreak in a short time period. This co-circulation highlighted the need to improve surveillance in order to prevent future potential severe dengue cases through antibody-dependent enhancement (ADE). Interestingly, it also proved the reliability and usefulness of the MBS-Lab for expedient outbreak response at the point of need, which allows early cases management.

## 1. Introduction

Dengue virus (DENV), a mosquito-borne virus, is the most important arboviral pathogen in tropical and subtropical regions throughout the world, with nearly one-third of the global human population at risk of infection [1]. Since 1950, DENV has emerged globally, infecting around 400 million people annually [2]. The virus has a positive single-strand RNA, belonging to the genus *Flavivirus*, *Flaviviridae* family [3].

Dengue encompasses a high variety of clinical syndromes ranging from flu like symptoms to severe disease [4] with a case fatality rate of 1–5% [5].

Two distinct cycles of virus transmission are described: a sylvatic cycle involving the arboreal mosquitoes and non-human primates, and an epidemic cycle involving human and domestic/peridomestic mosquitoes [6]. *Aedes* mosquitoes, mainly the *Aedes aegypti*, are the main vector of Dengue outbreaks worldwide [7]. The virus exists in four antigenically distinct serotypes, sharing around 65% genome similarity that is subdivided in phylogenetically distinct genotypes. The Asian region accounts for 70% of all reported cases [2]. Interestingly, all dengue serotypes were reported in Asian countries such as Malaysia where they cause regular outbreaks.

The epidemiology of dengue is not well understood in Africa and the virus is thought to be rare [8]. This underestimation is mainly due to the lack of surveillance, the presence of others prevalent febrile diseases as malaria, and a lack of diagnostic tools [9]. However, studies suggest that dengue exist in the continent as far back as 1926 [10]. Many epidemics were reported in Zanzibar, Burkina Faso, South Africa, and Senegal. Indeed, from 1960 to 2010, twenty laboratories confirmed dengue outbreaks in 15 different countries across the continent [8]. More recently, many African countries experienced outbreaks, such as Angola in 2013, Burkina Faso in 2016, and Côte d'Ivoire in 2017 [11, 12]. Many factors, including unplanned urbanization and climatic changes, are likely to shape the worldwide emergence by favouring vector infestation [13].

In Senegal, Dengue was first isolated in the Bandia area in 1970 [14]. After that, several studies have focused on the epidemiology of dengue fever. Since the 1980s, a few cases of dengue caused by DENV serotypes 2 and 4 have been reported [14, 15]. A particular interest has been the endemic circulation of DENV-2 in the southeastern region of the country. Virological and entomological surveillance data revealed epizootic diseases and few human cases. DENV-2 exhibits a cycle of sylvatic transmission in forest areas involving primates and wild mosquitoes of the genus *Aedes* (*Ae. luteocephalus*, *Ae. taylori*, and *Ae. furcifer*) [16]. Periods of epizootics of DENV-2 alternating with silent periods of 8 to 9 years were noted in this region in 1981, 1990, and 1999 [16]. In 1999, DENV-2 was isolated for the first time from *Ae. aegypti* [17], the main previously known vectors of DENV-2 being *Ae. furcifer* and *Ae. luteocephalus*. Isolation of DENV-2 from *Ae. aegypti* indicates the risk of dengue epidemics in this region. The sylvatic cycle of DENV-2 has also been detected in other West African countries: Côte d'Ivoire, Burkina Faso, Guinea, and Nigeria [15, 16, 18]. In Senegal, the sylvatic cycle has only been observed for DENV-2, whereas in other countries, particularly in Asia, the serotypes DENV-1 and DENV-4 were also involved [19].

Dengue fever re-emerged in Senegal with DENV-3 epidemic in 2009, a serotype that has never been detected before in Senegal or West Africa. A study conducted in 2010 indicated that DENV-3 was found in a Senegalese patient with signs of haemorrhagic dengue fever returning to Italy [20]. The emergence of DENV-3 has also been observed in other African countries including Cape Verde in 2009, where it caused the first ever-recorded dengue epidemic in the area [4]. Serotype 1, possibly, was circulating; in 1979, when two strains were isolated from two cases and serological studies showed 21.5% prevalence of anti-DENV-1 antibodies in Bandia, western part of Senegal. A high prevalence of DENV-1 antibody was also reported between 1981 and 1982 in Touba, Kaolack, and Mekhe during chikungunya virus (CHIKV) and yellow fever virus (YFV) epidemics. Serotype 4 was only reported in Senegal in circumstances that remain to be elucidated [21].

In September 2017 at Louga City, Northwestern Senegal, an outbreak of unexplained febrile cases occurred. Samples were shipped for laboratory diagnosis, at the WHO reference Collaborating Center (WHOCC) for arbovirus at Institut Pasteur de Dakar (IPD) for diagnosis, and test results revealed a circulation of DENV. The confirmation was followed by the deployment of a Mobile Biosafety Laboratory (MBS-Lab) for an epidemiological and virological field investigation of the outbreak between September 05 and December 17, 2017.

This paper describes the outbreak investigation and results from a field molecular investigation of suspected DENV cases with the use of point of need truck-based laboratory, as well as circulating serotypes and the phylogenetic characterization of the isolated strains.

## 2. Materials and Methods

### 2.1. Outbreak Notification and Mobile Biosafety Laboratory (MBS-Lab) Deployment

**Figure 1:**
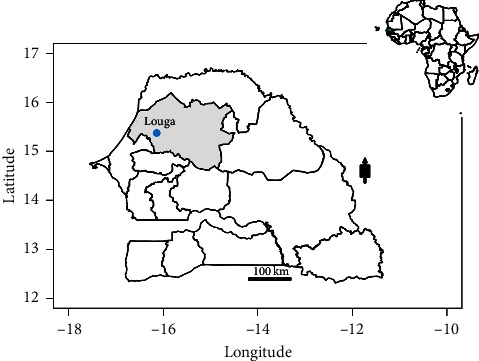
Map showing Louga City in Northwestern Senegal. Construction of the map was done using the maptools package installed in R studio version 1.2.1335; the shapefiles were downloaded from the free domain of the Geographic Information System (http://www.diva-gis.org).

Following the notification of high numbers of febrile cases as part of ongoing Syndromic Sentinel Surveillance network in Senegal (4S network) [22], a subset of 53 malaria negative blood samples, that were tested by Rapid Diagnostic Test (RDTs), were collected from Louga City in Northwestern Senegal (15°39′N, 16°21′W) (Figure 1) between 05 September 2017 and 10 October 2017. The samples were shipped to the WHOCC for arboviruses and haemorrhagic fever viruses at the Institut Pasteur de Dakar (IPD) to investigate a probable arboviral etiology. RNA was extracted from sera and tested for 5 arboviruses including dengue virus (DENV), chikungunya virus (CHIKV), yellow fever virus (YFV), Rift Valley fever virus (RVFV), and Zika virus (ZIKV) by qRT-PCR [23] from which only DENV gave a positive result.

In order to contain the increasing number of febrile cases, which required daily testing and early reporting of dengue cases, a mobile truck laboratory was deployed to the field in October 24 from the arboviruses and haemorraghic fever viruses lab located at IPD following a request from the Senegalese Ministry of Health (MoHS).

Indeed, this dengue outbreak was an opportunity to test the reliability and evaluate the use the truck-based MBS-Lab [24] for the molecular detection of dengue virus among suspected patients, directly in the field, without the need to ship the collected samples to the reference laboratory. The MBS-Lab was built in Belgium by the Praesens Foundation [24] and brought to the IPD, Dakar, Senegal, in September.

**Figure 2:**
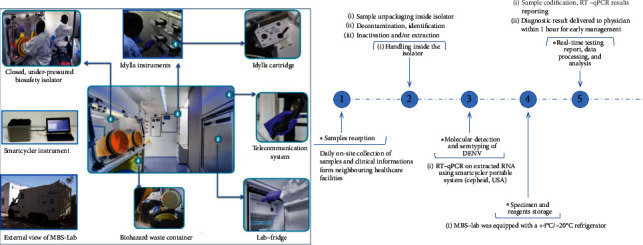
Mobile Biosafety Laboratory (MBS-Lab) workflow. (a) View and internal organization of MBS-Lab. (b) Workflow of dengue fever outbreak investigation.

The MBS-Lab houses a biosafety isolator with negative pressure and therefore provides a safe environment for handling different classes of pathogens, thus eliminating the need for personal protective equipment required for high containment laboratory facilities [24]. In addition, a real-time PCR testing system for DENV detection and serotyping was performed on a Smartcycler device (Cepheid, Sunnyvale, California, USA) integrated in the MBS-Lab, following a specific workflow (Figure 2).

### 2.2. Patients Selection

According to the standard WHO guidelines for case definitions [4], clinically suspected non-severe dengue fever (NSD) (with or without warning signs) is defined by acute fever (>38.5°C) and at least two of the following symptoms: severe headache, retro-orbital pain, nausea, vomiting, muscle and joint pains, rash, or leukopenia, while severe dengue (SD) is defined as NSD combined with bleeding, plasma leakage, and organ failure [25]. In collaboration with physicians from healthcare centers across Louga City, all the patients seeking medical care for febrile illnesses or history of fever lasting 2 to 7 days and that meet the case definition were enrolled and 5 ml venous blood sample was collected into a dry tube. For each patient, a standardized interview form was completed with clinical manifestations and demographic data. Any subject that meets case definition and positive by DENV qRT-PCR is defined as a confirmed case.

### 2.3. RNA Extraction

A total of 960 sera from eligible patients (suspected cases; any subject that meets clinical criteria) were obtained across Louga region. Viral RNA was extracted from 140 *μ*l of human sera using QIAmp viral RNA kit (Qiagen, Hilden, Germany) according to the manufacturer's instructions. RNA was eluted in 60 *μ*l of elution buffer.

### 2.4. RT-qPCR Detection

To confirm the dengue infection among suspected cases and defining the serotypes, respectively, a real-time RT-qPCR was directly performed in the field on the extracted RNA samples. The real-time molecular detection and serotyping were done using, respectively, the pan-Dengue primers described by [26] and TibMolBiol Modular Dx Dengue typing kit (Cat-No. 40-0700-24) both with the Quanta toughMix using a SmartCycler® II (Cepheid-USA) powered by the MBS-Lab. Briefly, dengue virus detection was performed using the following temperature profile: reverse transcription (RT) at 50°C for 10 min, activation at 95°C for 5 min, and 40 cycles of 2-step PCR at 95°C for 15 sec and 60°C for 30 sec. This optimised protocol allows for the detection of positive cases in less than one hour. All samples with a cycle threshold (Ct) value within the defined cut-off (Ct < 35) were considered as positive.

### 2.5. Sequencing and Phylogenetic Analysis

Random selection of samples of pre-defined serotypes was used for Sanger sequencing. For cDNA synthesis, 10 *μ*l of viral RNA was mixed with 1 *μ*l of the random hexamer primer (2 pmol) and the mixture was heated at 95°C for 2 min. Reverse transcription was performed in a 20 *μ*L reaction mix containing mixed of 2.5 U RNasin (Promega, Madison, USA), 1 *μ*L of deoxynucleotide triphosphate (dNTP) (10 mM each DNTP), 5 U of AMV reverse transcriptase (Promega, Madison, USA) by incubating at 42°C for 60 min. PCR products were generated using sets of primers described by [27, 28] which allow amplifying overlapping fragments of full *E* gene of DENV-1 and DENV-2. Five microliters of cDNA was mixed with 10 *μ*L of 10× buffer, 3 *μ*l of each primer, 5 *μ*l of dNTPs10 mM, 3 *μ*l of MgCl2, and 0.5 *μ*l of GoTaq polymerase (Promega, Madison, USA). The obtained amplicons were purified using a QIAquick Spin PCR Purification kit (Qiagen) and submitted for bidirectional sequencing then sent for bidirectional sequencing using an ABI 377 automated sequencer (Applied Biosystems) using the same PCR primers. Sequences obtained were merged using EMBOSS Merger software and the final results were analyzed using the Basic Local Alignment Tool (BLAST, http://www.ncbi.nlm.nih.gov/). Nucleotide sequences alignment was generated using the ClustalW algorithm implemented in Mega version 6 [29].

Representative DENV-1 and DENV-2 were downloaded from GenBank and aligned with the obtained sequences using ClustalW algorithm implemented in Mega software. A maximum likelihood (ML) tree was inferred for each serotype using IQ-tree [30].

### 2.6. Ethical Consideration

The Senegalese National Ethical Committee of the Ministry of Health approved the surveillance protocol as a less than minimal risk research, and written consent forms were not required. Oral consent to participate was obtained from all patients or parents/guardians of minors included in this study as required by the Senegalese National Ethical Committee of the Ministry of Health. Throughout the study, the database was shared with the Epidemiology Department at the Senegalese Ministry of Health and Prevention for appropriate public health action.

### 2.7. Statistical Analysis

The demographic and clinical characteristics of the study population between suspected and confirmed dengue cases were analyzed using *χ*^2^-test or Fisher's exact test. Mean age and mean sampling delay comparison between suspected and confirmed dengue cases were done using Kruskal–Wallis test. A *p* value <0.05 was considered statistically significant. Prevalence of DENV was calculated for each modality.

## 3. Results

### 3.1. Demographic Characteristics and Epidemiology

**Figure 3:**
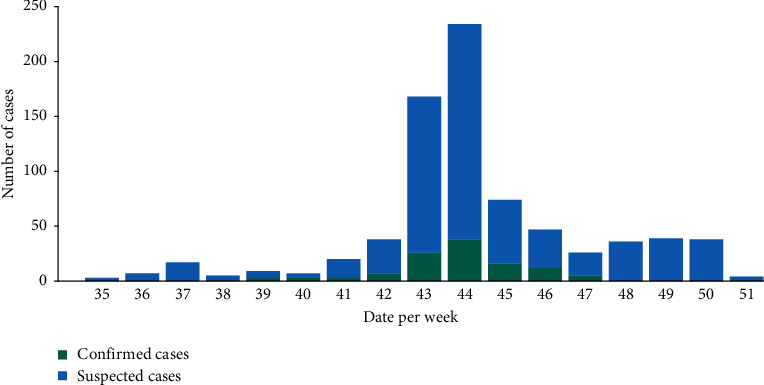
Distribution of suspected and confirmed DENV human cases according to epidemiological weeks in Louga City, 2017.

From September 05 to December 17, 2017, a total of 960 serum samples were collected in 8 localities across the Louga City from patients who were suspected to have DENV infections. The outbreak began at epidemiological week 39 and ended at week 47; the highest number of confirmed DENV cases was recorded during week 44 (Figure 3). Most samples were collected from various districts (DS) in Louga City including DS Louga (*n* = 485), DS Darou Mousty (*n* = 197), and DS Dahra (*n* = 125). The overall infection rate of DENV was 13.64% (131/960). Among 8 localities that reported at least 1 suspected case, no confirmed cases were recorded in DS Kebemer, DS Linguere, and DS Sakal. For the confirmed cases, 80.15% (*n* = 105/131) was from Louga district and 9.16% (*n* = 12/131) from DS Darou Mousty while the least number was recorded in Koki with only two confirmed cases (1.52%) (Table 1). Among the suspected dengue fever cases, 447 (46.52%) were males (*M*) and 513 (53.43%) were females (F). M : F sex ratio for the positive cases was 1.14 (70 males, 61 females). There was no significant DENV-1 positivity rate difference according to gender (*p*=0.09; Pearson's *χ*^2^-test) (Table 1). Table 1 shows the overall repartition of cases according to the age group. Most of confirmed cases belonged to 10- to 20-year-old age groups (47.6%) followed by the 20–30 years (27.8%), with the lowest positivity rate to dengue virus recorded in age group 40–50 years (3.2%). The rate of confirmed DENV cases varied significantly according to age group (*p* < 0.0012; Pearson's *χ*^2^-test).

### 3.2. Clinical Presentations

During the outbreak of DENV infection, fever was the clinical presentation recorded in all suspected cases followed by headaches (90%). The other common clinical features were arthralgia (61.66%) and myalgia (44.89%) (Table 1).

### 3.3. Circulating Dengue Virus Serotypes

To define the circulating serotypes, 104 dengue positives samples were subjected to serotyping using real-time RT-qPCR (Table 2). We found DENV-1 (100/104; 96.2%) as the dominant serotype during this outbreak, followed by DENV-2 (04/104; 3.8%). No cases of DENV-3 or DENV-4 were found.

### 3.4. Phylogenetic Inference

**Figure 4:**
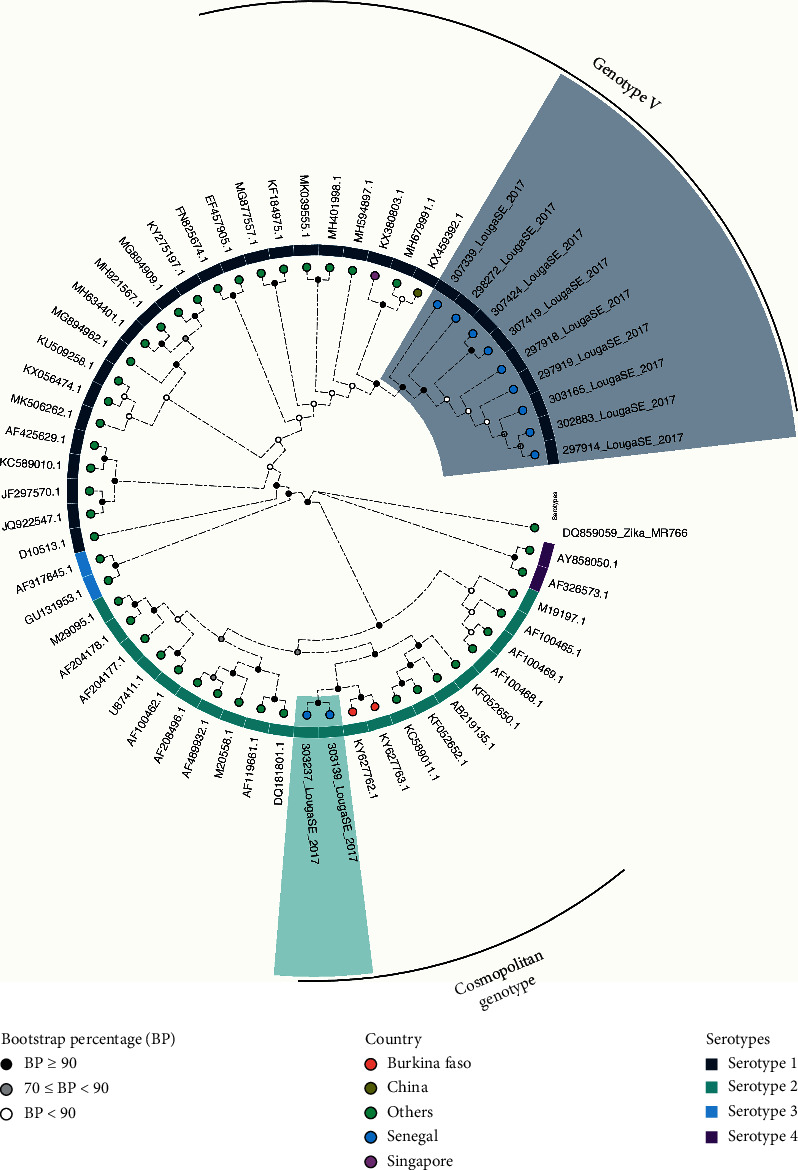
Phylogenetic analysis of complete E gene of the Louga City outbreak isolates. The analysis was run using an alignment representing the described dengue virus serotype 1 and 2 genotypes, in addition to the Louga strains highlighted, respectively, in light blue (serotype 1) and light green (serotype 2). The maximum likelihood phylogeny applying the TIM2 + *F* + *R*8 model was generated using IQ-tree, and tree support was calculated after 1000 bootstrap replicates.

Phylogenetic analysis of the complete *E* gene of a subset of both dengue serotypes 1 and 2 that were detected during the outbreak showed that the DENV-2 isolate clustered with the strain from the 2016 outbreak in Burkina Faso (KY627777) and belonged to the cosmopolitan genotype. On the other hand, the DENV-1 serotype clustered with the strain that was isolated in Singapore (KX380803) in 2013 belonging to the genotype V (Figure 4).

## 4. Discussion

Worldwide, DENV infection occurs in an estimated 50 million people annually, making it the most common mosquitoes-borne virus in the world [4]. Approximately 8% to 100% of the cases result in symptomatic infections [31]. The fatality rate of this arboviral infection is around 1% [32] and can be considerably reduced by early diagnostic guiding appropriate management [33]. Little information is available on the impact of DENV among Senegalese populations and the mechanisms involved in the emergence of DENV outbreaks. The current study reports an epidemic of DENV serotypes 1 and 2 in Senegal 8 years after the last reported dengue fever epidemic caused by the serotype 3 [34]. This epidemic was monitored over 16 weeks, with 960 suspected cases, out of which 131 were confirmed. Dengue infection cases were predominant in individuals aged between 10 and 20 years (47.6%); however, equal infection rates were observed in males and females (*p*=0.09; Pearson's *χ*^2^-test). Similar trends were observed in South America by Günther and colleagues [35] and in a study conducted by Chang and colleagues in Taiwan [36]. Several studies conducted in Cambodia, Sri Lanka, Singapore, and Philippines [37–39] reported that males seem to be more prone to DENV infections than females, which was probably due to more outdoor work habits of males which increases their chance of being bitten by mosquitoes [40]. Studies involving large sample sizes, and combining molecular and serological investigations, are needed to confirm this possible gender difference in infection rate.

The most frequent symptoms observed were fever, headaches, arthralgia, and myalgia. The same clinical manifestations trends were found in a study conducted in Malaysia [41]. Otherwise, fever is known to be the most common symptom leading patients to seek healthcare [42]. Indeed, acute febrile episodes are caused by various bacterial, parasitic, and viral pathogens, and infections with these agents result in patients presenting with dengue fever-like symptoms [43]. The differential diagnosis of the aetiologic agent of the fever is hampered by the lack of accurate, rapid, and affordable diagnostic techniques in low resourced settings [44, 45]. The lack of awareness and effective surveillance indicates that dengue fever is likely to be under-recognized and under-reported in Africa [8]. Returning travellers from Africa to Europe are effective sentinels for the detection of unreported outbreaks occurring abroad [46]. In October 2009, a case of DENV-3 was recorded in Turin, Italy, from a patient returning from Louga City [34, 47]. To overcome this gap on differential diagnosis, capabilities are urgently needed and constitute a critical step in assessing the incidence of dengue fever in Africa. A recent study conducted on aetiologies of non-malarial febrile illnesses using a mobile laboratory, based on recombinase polymerase amplification (RPA), allowed the molecular detection of three sporadic of DENV-1 cases in the suburb of Dakar [23]. Also, a previous study conducted during the DENV-3 urban epidemic in Senegal in 2009 revealed that the Louga region had a high density of *Ae. aegypti* mosquitoes [34], which constitute the main vector for DENV in tropical and subtropical areas [48]. This fact, combined with population growth and unplanned urbanization, makes a risk factor for dengue fever outbreak occurrence in the region.

This field investigation was also an interesting opportunity to test the role of MBS-Lab for timely outbreak investigation response in an area located about 200 km away from the reference laboratory. For most of the previous outbreak investigations, the turnaround time to obtain results can take up to several days. Louga area is located 222,8 km from the World Health Organization (WHO) Reference Center for Arboviruses and haemorraghic fever viruses at the Institute Pasteur in Dakar (IPD); shipping of samples took about 5 hours and this needs to be performed daily to allow timely diagnosis of new suspected dengue cases and the results to be available in at least 24 hours. In this study, the MBS-Lab was installed in order to receive samples collected around Louga City within 1 hour for immediate processing and the results sent within the next 2 hours after the sample were received.

Our molecular setup allowed the detection of dengue fever cases within 1 hour (sample pre-processing and extraction step not considered), and the direct report of the results to the local healthcare worker for early case management, which is known to improve outcome.

The serotyping performed on the field showed the predominance of DENV-1 (100/104) and DENV-2 in few cases (04/104), with no co-infections among the patients studied. Co-infection, with more than one serotype, is likely to be much higher when multiple dengue serotypes co-circulate in a given population [49], and this poses a risk of emergence of recombinant virus strains that could have distinct properties [50].

Studies in Gabon [51] and in India [49, 52] revealed that regions where simultaneous circulation of more than one serotype is observed are more prone to severe DENV infections. In view of the risk discussed above, early detection of circulating serotypes through molecular surveillance is crucial for control and preparedness.

Phylogenetic analysis of isolates suggests that they belonged, respectively, to DENV-1 genotype V and DENV-2 cosmopolitan genotype. The strains were closely related to isolates detected in Singapore 2013 [53] and during the DENV-2 outbreak in Burkina Faso [54]. Hashimoto and colleagues [55] reported imported DENV-2 cases linked to Burkina Faso to Japan and highlighted the spread of Burkina Faso strain to other parts of the world [56].

Our study also highlights the spread of the Burkina Faso strains within Africa. This finding suggests the co-circulation of two DENV serotypes during the same outbreak and suggests that an increased urban DENV activity is plausible in Senegal as suggested by Faye and Colleagues [34], even if no DENV-1/DENV-2 co-infection was reported during this outbreak. Surveillance and control measures after reported cases in Louga are needed to prevent propagation of DENV in neighboring regions.

### 4.1. Limitations of the Study

Overall, during this DENV outbreak investigation at Louga City, we evaluate for the first time in Senegal a truck-based mobile laboratory for epidemic response purpose. This constitutes an important and relevant public health tool with positive impact on early cases management and diagnosis at the point of need. Despite the reliability of the MBS-Lab, some limitations associated with the study need to be mentioned. First, during the study we only focused on molecular detection of DENV RNA among suspected cases, but no NS1 antigen detection and non-IgM antibody were assessed on the suspected cases. Known to provide a wide window for DENV infections diagnosis, NS1 antigen detection is a good biomarker of acute illness. In this context, negative DENV PCR does not mean an absence of infection and could lead to a possible underestimation of the actual number dengue fever cases during the epidemic. Interestingly, the combination of NS1 detection and IgM detection is shown to have the potential to outperform PCR diagnosis. Second, during the outbreak investigation, only the DENV was targeted, but a high number of febrile cases that are not due to DENV (829/960; 86.35%) indicate probable co-circulation of other pathogens and call for systematic differential diagnostics or a syndromic approach to avoid cryptic outbreaks that are caused by viruses with similar clinical signs.

## Figures and Tables

**Table 1:** Epidemiologic and clinical characteristics of suspected and confirmed dengue fever cases, Louga, Senegal, 2017.

	Negatives (*n* = 829)	Positives (*n* = 131)	Total (*n* = 960)	*p* value
Age				0.149^2^
Median	22	20	21	
Q1, Q3	12, 33	15, 29.75	13, 32	
Age group				<**0.001**^1^
N-Miss	36	05	41	
[0, 10]	174 (21.9%)	7 (5.6%)	181 (19.7%)	
[10, 20]	207 (26.1%)	60 (47.6%)	267 (29.1%)	
[20, 30]	178 (22.4%)	35 (27.8%)	213 (23.2%)	
[30, 40]	120 (15.1%)	20 (15.9%)	140 (15.2%)	
[40, 50]	58 (7.3%)	4 (3.2%)	62 (6.7%)	
[50, 90]	56 (7.1)	0 (0.0%)	56 (6.1%)	
Sex				0.090^1^
F	452 (54.5%)	61 (46.6%)	513 (53.4%)	
M	377 (45.5%)	70 (53.4%)	447 (46.6%)	
Arthralgia				**<0.001** ^1^
No	341 (41.1%)	27 (20.6%)	368 (38.3%)	
Yes	488 (58.9%)	104 (79.4%)	592 (61.7%)	
Asthenia				**0.022** ^1^
No	475 (57.3%)	61 (46.6%)	536 (55.8%)	
Yes	354 (42.7%)	70 (53.4%)	424 (44.2%)	
Headache				0.110^1^
No	88 (10.6%)	8 (6.1%)	96 (10.0%)	
Yes	741 (89.4%)	123 (93.9%)	864 (90.0%)	
Retro-orbital pain				0.159^1^
No	825 (99.5%)	129 (98.5%)	954 (99.4%)	
Yes	4 (0.5%)	2 (1.5%)	6 (0.6%)	
Rash				0.961^1^
No	823 (99.3%)	130 (99.2%)	953 (99.3%)	
Yes	6 (0.7%)	1 (0.8%)	7 (0.7%)	
Myalgia				**0.013** ^1^
No	470 (56.7%)	59 (45%)	529 (55.1%)	
Yes	359 (43.3%)	72 (55%)	431 (44.9%)	
Outcome				0.658^1^
Died	10 (1.2%)	1 (0.8%)	11 (1.1%)	
Living	819 (98.8%)	130 (99.2%)	949 (98.9%)	
Symptoms-sampling delay				0.083^2^
Median	2.000	1.000	2.000	
Q1, Q3	1.000, 3.000	1.000, 2.000	1.000, 2.000	
Medical structure				<**0.001**^1^
	15 (1.8%)	0 (0.0%)	15 (1.6%)	
DS Dahra	117 (14.1)	8 (6.1%)	125 (13%)	
DS Darou Mousty	185 (22.3%)	12 (9.2%)	197 (20.5)	
DS Kebemer	19 (2.3%)	0 (0%)	19 (2.0%)	
DS KMS	41 (4.9%)	4 (3.1%)	45 (4.7%)	
DS Koki	15 (1.8%)	2 (1.5%)	17 (1.8%)	
DS Linguere	28 (3.4%)	0 (0.0%)	28 (2.9%)	
DS Louga	380 (45.8%)	105 (80.2%)	485 (50.3%)	
DS Sakal	29 (3.5%)	0 (0.0%)	29 (3.0%)	

Significant *p* values are indicated in bold. “1” stands for *χ*^2^-test or Fisher's exact test. “2” stands for the Kruskal–Wallis test.

**Table 2:** Circulating serotypes of DENV among infected patients.

Molecular diagnostic	*n* (%)	95% CI
qRT-PCR DENV 1–4	131/960 (13.54)	11.43–15.87
DENV-1	100/104 (00)	90.44–98.74
DENV-2	04/104 (3.84)	1.05–9.55
DENV-3	00/104 (00)	NA
DENV-4	00/104 (00)	NA
Co-infections	00/104 (00)	NA

## Data Availability

The data used to support the findings of this study are available from the corresponding author upon request.
